# Multiple long-term conditions and their association with quality of life and healthcare utilisation among adults in India: a cross-sectional analysis of WHO SAGE Waves 2 and 3

**DOI:** 10.1136/bmjopen-2026-116399

**Published:** 2026-08-11

**Authors:** Amrit Banstola, Sayem Ahmed, M Swathi Shenoy, Priti Gupta, Dimple Kondal, Radhika Bhagat, Shifalika Goenka, Kamlesh Khunti, Dorairaj Prabhakaran, Sailesh Mohan, Subhash Pokhrel

**Affiliations:** 1 Department of Health Sciences, Brunel University London, Uxbridge, UK; 2 Department of Community Medicine, Kasturba Medical College Mangalore, Mangaluru, Karnataka, India; 3 Centre for Chronic Disease Control, New Delhi, Delhi, India; 4 University of Leicester, Leicester, UK

**Keywords:** India, Multimorbidity, Quality of Life

## Abstract

**Abstract:**

**Objectives:**

To examine the prevalence and disease-combination patterns of multiple long-term conditions (MLTCs) among adults in India and their associations with quality of life and healthcare utilisation.

**Design:**

Cross-sectional analysis of two nationally representative survey waves.

**Setting:**

Community-based household survey conducted across six Indian states (Assam, Karnataka, Maharashtra, Rajasthan, Uttar Pradesh and West Bengal), part of the WHO Study on global AGEing and adult health.

**Participants:**

9116 adults aged 18 years and older in Wave 2 (2015) and 7885 adults in Wave 3 (2019–2020).

**Primary and secondary outcome measures:**

Quality of life, assessed with the 8-item WHO Quality of Life scale (WHOQoL-8, range 0–100), was the primary outcome. Outpatient visits and hospitalisations in the preceding 12 months were the secondary outcomes. Associations with MLTC were estimated using Tobit regression for quality of life and Poisson regression for healthcare utilisation, adjusting for sociodemographic, socio-economic and behavioural factors.

**Results:**

MLTC prevalence rose from 19.2% to 24.4% between waves and was highest among adults aged 60 years and older, women, urban residents and the wealthiest quintile. Hypertension–cataracts, hypertension–arthritis and hypertension–diabetes were the most prevalent dyads. Depression–chronic obstructive pulmonary disease (COPD) and depression–stroke combinations had the poorest quality of life. Physical and functional domains declined most as MLTC increased, while the financial domain remained stable. Compared with adults without chronic conditions, WHOQoL-8 scores fell in a stepwise pattern with one, two and three or more conditions (–2.6, –5.1 and –6.7 points in 2015; –3.3, –5.6 and –7.9 in 2019–2020; all p<0.001). The COPD–stroke combination had the highest outpatient use, and depression–angina the highest hospitalisation rate. Healthcare use rose incrementally with condition count (p<0.001).

**Conclusions:**

MLTC are rising in India and are associated with poorer quality of life and higher healthcare use. High-burden combinations, particularly depression–COPD, depression–stroke, COPD–stroke and depression–angina, warrant targeted interventions to improve quality of life and manage rising healthcare demands.

**STRENGTHS AND LIMITATIONS OF THIS STUDY**

- Two large, nationally representative waves of the WHO Study on global AGEing and adult health (SAGE) survey in India (Waves 2 and 3) were analysed.
- Quality of life was assessed using the validated 8-item WHO Quality of Life instrument alongside measures of healthcare utilisation.
- Tobit regression modelled the censored quality of life outcome, and Poisson regression modelled the count-based healthcare utilisation outcome, adjusting for a wide range of sociodemographic, socio-economic and behavioural covariates.
- Disease conditions and healthcare use were self-reported, which may introduce recall bias.
- The cross-sectional design of each wave prevents causal inference, and the nine self-reported conditions captured by SAGE understate the true burden of multiple long-term conditions.

## Introduction

 The coexistence of two or more chronic conditions, known as multiple long-term conditions (MLTC) or multimorbidity,^1^ is an urgent and growing global health challenge, driven by population ageing and the rising burden of non-communicable diseases (NCDs).^2 3^ Recent meta-analyses estimate the global pooled prevalence of MLTC at between 37.2% (95% CI 34.9% to 39.4%)^4^ and 42.4% (95% CI 38.9% to 46.0%),^5^ with the highest rates observed in South America (45.7%), North America (43.1%), Europe (39.2%), Asia (35.0%) and Africa (28.2%).^4^ Substantial variation in prevalence estimates reflects methodological differences in defining and counting conditions.^5^

Low- and middle-income countries (LMICs) are experiencing particularly rapid growth in MLTC, often with onset at younger ages than in high-income countries. This pattern reflects multiple interacting factors, including accelerated epidemiological transitions, physiological differences, epigenetic and early-life influences, and underlying health system vulnerabilities, despite at times a lower prevalence of conventional risk factors.^6–11^ In India, which accounts for more than one-sixth of the global population, the prevalence of MLTC varies widely depending on study population, age group and definition used, ranging nationally from roughly 7% to 24% in adults,^12–15^ with higher figures in specific patient groups or older adults.^16–20^ Hypertension, diabetes, cataracts and arthritis are consistently the most common conditions, influenced by sociodemographic risk factors such as age, sex, wealth, education and urbanisation.^13 21^

Despite the growing burden, evidence on health-related quality of life (HRQoL) and healthcare utilisation in India remains limited and methodologically constrained.^22–24^ Existing HRQoL studies are predominantly cross-sectional, conducted in primary care or community settings within a single state.^25–27^ Some have relied on proxy measures such as self-rated health, mobility and life satisfaction rather than validated instruments such as EuroQol five-dimensional questionnaire (EQ-5D), the 12-Item Short-Form Health Survey (SF-12), WHO Quality of Life (WHOQoL), restricting comparability with international evidence.^28^ Other India-specific findings are embedded within cross-country analyses using the WHO Study on global AGEing and adult health (SAGE) Wave-1 data (2007–2010), which is now outdated.^29 30^ One recent study used a large cross-sectional dataset but was limited to older adults aged 60 years and above, further narrowing its generalisability.^15^ Qualitative evidence further demonstrates that living with MLTC in India adversely affects quality of life, functional capacity and daily living.^31^ Evidence on healthcare utilisation is often based on adults recruited from primary care facilities in a single state with limited generalisability.^32 33^ Studies have largely measured utilisation in terms of hospitalisation^34^ or measured only through out-of-pocket expenditures,^35^ while others target specific subgroups such as those with complex multimorbidity^36^ or diabesity.^37^

To address these gaps, the present study uses two recent, nationally representative waves of SAGE India (2015 and 2019–2020) to:

1. Estimate MLTC prevalence, patterns and disease combinations.
2. Examine associations between MLTC, HRQoL and healthcare utilisation (outpatient visits and hospitalisation).
3. Assess how these associations vary by disease combination and socio-economic, demographic and behavioural characteristics.

By providing nationally representative evidence, this study seeks to inform the development of targeted strategies for managing the growing burden of MLTC in India.

## Methods

### Data source

The SAGE is a longitudinal household survey and is part of a multicountry project conducted in China, Ghana, India, Mexico, Russia and South Africa.^38^ This study used anonymous secondary data from two latest waves of the SAGE conducted in 2015 and 2019–2020 in India. Wave 2 (2015) included a total of 9116 adults, with a response rate of 77%, while Wave 3 (2019–2020) included 7885 adults, with a response rate of 89%. The study employed a multistage stratified cluster random sampling design to ensure representative observations. The SAGE Waves 2 and 3 were implemented in six selected Indian states: Assam, Karnataka, Maharashtra, Rajasthan, Uttar Pradesh and West Bengal, to achieve a nationally representative sample of older adults aged ≥50 years and a smaller cohort of adults aged 18–49 years. Although data collection was limited to these states, the sampling strategy was designed to ensure national representativeness. A detailed description of the study including sample design is provided elsewhere.^39 40^

### Outcome variables

Quality of life: The 8-item WHOQoL (WHOQoL-8) instrument was used to quantify quality of life and included two questions in each of four broad domains: physical, psychological, social and environmental.^41^ The WHOQoL-8 is a concise version of the WHOQoL instrument, designed to assess an individual’s subjective quality of life across key domains with only eight items. It is a simplified yet valid tool, derived from the WHOQoL-BREF (brief version) to cover broad aspects of quality of life.^42^ Quality of life was assessed by asking respondents to rate their satisfaction with eight domains of their lives, namely (1) overall quality of life, (2) overall health, (3) energy levels, (4) ability to perform daily activities, (5) self-esteem, (6) personal relationships, (7) money and (8) living conditions. The scores for each domain in the WHOQoL are determined using a five-point response scale, ranging from very satisfied to very dissatisfied. A composite score was created by summing the responses across the different questions and rescaling the result from 0 to 100 where a higher score indicated better quality of life.

Healthcare utilisation: Healthcare utilisation was measured in terms of outpatient visits and hospitalisation. Frequency of outpatient visits was measured as the number of times a participant had an outpatient visit in the preceding 12 months. Hospitalisation was measured as any overnight stays in the hospital that lasted for at least one night in the past 12 months.

### Independent variables/covariates

Independent variables included sociodemographic characteristics (sex, age, marital status, ethnic group, religion, education, place of residence), economic status (wealth, health insurance), behavioural factors (physical activity, tobacco use, alcohol consumption) and body mass index (BMI). Sex was categorised as male or female, and age into 18–44 years, 45–59 years and ≥60 years, corresponding to the standard elderly cut-off in India. Marital status was grouped as never married, currently married/cohabiting, separated/divorced or widowed. Ethnic group was classified as scheduled tribe, scheduled caste, other backward caste, none of the above or others, and religion as Hinduism, Islam or others. Education was measured in years of schooling (none, 1–5, 6–9 or ≥10 years). Wealth status was categorised into quintiles based on household assets: lowest, second, middle, fourth and highest. Health insurance was recorded as yes or no, and place of residence as rural or urban.

BMI was classified using WHO cut-offs.^43^ Physical activity level was assessed via the International Physical Activity Questionnaire and classified as low, moderate or high following standard Metabolic Equivalent of Task (MET-minute) thresholds. Tobacco use was categorised as never, current or past. Alcohol consumption was categorised as person who abstains from alcohol for life, person who does not drink heavily, person who infrequently drinks alcohol heavily or person who frequently drinks alcohol heavily. Detailed definitions for these covariates are provided in the supplementary file (online supplemental table S1).

MLTC was defined as the simultaneous presence of two or more chronic conditions in an individual.^44^ For this study, nine self-reported chronic conditions including angina pectoris, arthritis, asthma, chronic lung disease, diabetes mellitus, hypertension, stroke, depression and cataracts were included.

### Statistical analysis

Descriptive statistics are presented as frequencies and percentages to report the characteristics of study population and prevalence. Means with SD for continuous variables (eg, quality of life scores). χ² tests were used to examine associations between MLTC prevalence and sociodemographic characteristics. One-way analysis of variance compared mean quality of life scores and healthcare utilisation across different numbers of chronic conditions, with equal variances assessed using Bartlett’s test.

The analysis used complete case analysis. Missing data were declared and participants with incomplete information on key variables were excluded from respective analyses. Multiple regression models examined the association between number of MLTC and outcome variables (eg, quality of life and healthcare utilisation), adjusting for potential confounders. Separate models were fitted for each wave. Tobit regression was used for quality of life outcomes to account for the bounded nature of WHOQoL-8 scores (0–100 scale) and potential ceiling and floor effects. Poisson regression models were applied to healthcare utilisation outcomes (outpatient visits and hospitalisations), appropriate for count data with non-negative integer values.

All models included number of MLTC dyads as the primary exposure (reference: zero conditions) and were adjusted for sociodemographic factors (sex, age, marital status, ethnicity, religion), socio-economic variables (education, wealth, health insurance, residence) and health behaviours (BMI, physical activity, tobacco use, alcohol consumption). Model fit was assessed using likelihood ratio χ² tests.

Results are reported as regression coefficients with 95% CIs. For quality of life models, negative coefficients indicate poorer quality of life; for healthcare utilisation models, positive coefficients indicate increased service use. We used 5% significance level for statistical tests. Analyses were conducted separately for Wave 2 and Wave 3 to explore temporal trends. Statistical analyses were performed using Stata V.17.0 (StataCorp, Texas, USA).

### Patient and public involvement

Patients or the public were not involved in the design, or conduct, or reporting, or dissemination plans of this research.

## Results

### Sample characteristics

The sample characteristics of SAGE Waves 2 and 3 are presented in table 1. In Wave 2, the mean age was 56.7 years (SD 14.5). Over half were female (54.3%), three-quarters were married or cohabiting (74.5%) and most identified as Hindu (83.8%). About 7.9% belonged to scheduled tribes, 45.8% had no formal schooling and 19.2% were in the lowest wealth quintile. Only 9.8% reported having health insurance, while 78.9% lived in rural areas. Obesity was observed in 3.7% of participants, 8.2% reported low physical activity, 28.2% used tobacco and 9.0% consumed alcohol.

**Table 1:** Sociodemographic characteristics and MLTC prevalence in Wave 2 and 3

	Wave 2			Wave 3		
	Total (%) n=9116	MLTC prevalence (%) n=1747 (19.2%)	P value	Total (%) n=7885	MLTC prevalence (%) n=1925 (24.4%)	P value
Sex						
Female	4946 (54.3)	974 (19.7)	0.16	4345 (55.1)	1114 (25.6)	0.005**
Male	4170 (45.7)	773 (18.5)		3540 (44.9)	811 (22.9)	
Age in years						
18–44 years	1521 (16.7)	66 (4.3)	<0.001***	853 (10.8)	27 (3.2)	<0.001***
45–59 years	3381 (37.1)	524 (15.5)		2673 (33.9)	490 (18.3)	
60+ years	4214 (46.2)	1157 (27.5)		4359 (55.3)	1408 (32.3)	
Mean (SD)	56.7 (14.5)			60.0 (13.1)		
Marital status						
Never married	494 (5.4)	24 (4.9)	<0.001***	257 (3.3)	11 (4.3)	<0.001***
Currently married/cohabiting	6788 (74.5)	1224 (18.0)		5866 (74.4)	1344 (22.9)	
Separated/divorced	50 (0.5)	11 (22.0)		44 (0.6)	10 (22.7)	
Widowed	1784 (19.6)	488 (27.4)		1718 (21.8)	560 (32.6)	
Ethnic group						
Schedule tribe	721 (7.9)	98 (13.6)	<0.001***	585 (7.4)	113 (19.3)	<0.001***
Schedule caste	1585 (17.4)	263 (16.6)		1351 (17.1)	256 (18.9)	
Other backward caste	4178 (45.9)	834 (20.0)		2805 (35.6)	682 (24.3)	
None of the above	1461 (16.0)	311 (21.3)		2874 (36.4)	808 (28.1)	
Others	1165 (12.8)	241 (20.7)		270 (3.4)	66 (24.4)	
Religion						
Hinduism	7633 (83.8)	1430 (18.7)	0.054	6541 (83.0)	1605 (24.5)	0.13
Islam	1123 (12.3)	240 (21.4)		875 (11.1)	223 (25.5)	
Others	356 (3.9)	77 (21.6)		469 (5.9)	97 (20.7)	
Years of schooling						
No formal schooling	4165 (45.8)	739 (17.7)	0.003**	3250 (41.3)	752 (23.1)	0.14
1–5 years	1568 (17.2)	345 (22.0)		1643 (20.9)	414 (25.2)	
6–9 years	1415 (15.5)	275 (19.4)		1289 (16.4)	336 (26.1)	
10+ years	1955 (21.5)	387 (19.8)		1696 (21.5)	420 (24.8)	
Wealth quintiles						
Lowest	1752 (19.2)	240 (13.7)	<0.001***	1468 (18.6)	220 (15.0)	<0.001***
Second	1727 (18.9)	275 (15.9)		1541 (19.5)	325 (21.1)	
Middle	1776 (19.5)	313 (17.6)		1572 (19.9)	366 (23.3)	
Fourth	1841 (20.2)	368 (20.0)		1607 (20.4)	438 (27.3)	
Highest	2020 (22.2)	551 (27.3)		1697 (21.5)	576 (33.9)	
Health insurance						
No	8225 (90.2)	1552 (18.9)	0.03*	5693 (72.2)	1362 (23.9)	0.1
Yes	891 (9.8)	195 (21.9)		2192 (27.8)	563 (25.7)	
Place of residence						
Rural	7192 (78.9)	1220 (17.0)	<0.001***	6417 (81.4)	1442 (22.5)	<0.001***
Urban	1924 (21.1)	527 (27.4)		1468 (18.6)	483 (32.9)	
Body mass index						
Underweight	2271 (27.1)	333 (14.7)	<0.001***	1476 (19.8)	278 (18.8)	<0.001***
Normal weight	4573 (54.6)	843 (18.4)		4169 (55.8)	918 (22.0)	
Overweight	1224 (14.6)	324 (26.5)		1399 (18.7)	446 (31.9)	
Obese	311 (3.7)	102 (32.8)		426 (5.7)	168 (39.4)	
Physical activity						
Low	750 (8.2)	186 (24.8)	<0.001***	497 (6.3)	163 (32.8)	<0.001***
Moderate	5126 (56.2)	1022 (19.9)		3837 (48.7)	1079 (28.1)	
High	3240 (35.5)	539 (16.6)		3551 (45.0)	683 (19.2)	
Tobacco use						
Never	6184 (67.8)	1143 (18.5)	<0.001***	4827 (61.2)	1193 (24.7)	<0.001***
Current	2574 (28.2)	502 (19.5)		2600 (33.0)	577 (22.2)	
Past	358 (3.9)	102 (28.5)		458 (5.8)	155 (33.8)	
Alcohol consumption						
Person who abstains from alcohol for life	8275 (91.0)	1565 (18.9)	0.031*	7241 (91.8)	1735 (24.0)	0.002**
Person who does not drink heavily	585 (6.4)	138 (23.6)		528 (6.7)	163 (30.9)	
Person who infrequently drinks alcohol heavily	74 (0.8)	11 (14.9)		22 (0.3)	8 (36.4)	
Person who frequently drinks alcohol heavily	159 (1.7)	33 (20.8)		94 (1.2)	19 (20.2)	

*p<0.05; **p<0.01; ***p<0.001.

P values from Pearson χ² tests comparing participants with and without MLTC within each wave.

Percentages are calculated among participants with non-missing data for each variable; missing data were as follows: ethnic group (n=6, Wave 2), religion (n=4, Wave 2), years of schooling (n=13, Wave 2; n=7, Wave 3), body mass index (n=737, Wave 2; n=415, Wave 3), and alcohol consumption (n=23, Wave 2).

MLTC, multiple long-term condition.

In Wave 3, distributions were broadly similar. The mean age increased to 60.0 years (SD 13.1); 55.1% were female and 74.4% were married or cohabiting. The proportion without formal schooling declined to 41.3%, while health insurance coverage rose to 27.8%. Rural residents comprised 81.4% of the sample, and 18.6% were in the lowest wealth quintile. Obesity increased slightly to 5.7%, low physical activity decreased to 6.3%, tobacco use rose to 33.0% and alcohol use was 8.2%.

### Prevalence of MLTC

In Wave 2, the prevalence of MLTC was 19.2%, with similar rates among men and women (18.5% vs 19.7%) (table 1). Prevalence increased steeply with age, reaching 27.5% among adults aged ≥60 years and was highest among widowed individuals. Socio-economic gradients were evident, with prevalence rising from 13.7% in the poorest to 27.3% in the richest quintile. Urban residents (27.4%) and obese individuals (32.8%) showed higher prevalence than their rural (17.0%) and normal weight (18.4%) counterparts. Those with low physical activity, past tobacco use or alcohol consumption also reported higher prevalence.

In Wave 3, MLTC prevalence increased to 24.4%, with significantly higher rates among women (25.6%) than men (22.9%) (table 1). Age-related differences persisted, with prevalence reaching 32.3% among those aged ≥60 years and was highest among widowed individuals. The socio-economic gradient became more pronounced, ranging from 15.0% in the lowest to 33.9% in the highest wealth quintile. Prevalence rose sharply with BMI, from 18.8% among underweight to 39.4% among obese individuals (p<0.001). Urban residents and those with low physical activity or past tobacco use again demonstrated elevated prevalence, consistent with patterns observed in Wave 2.

### Prevalence by number of disease count

**Figure 1:**
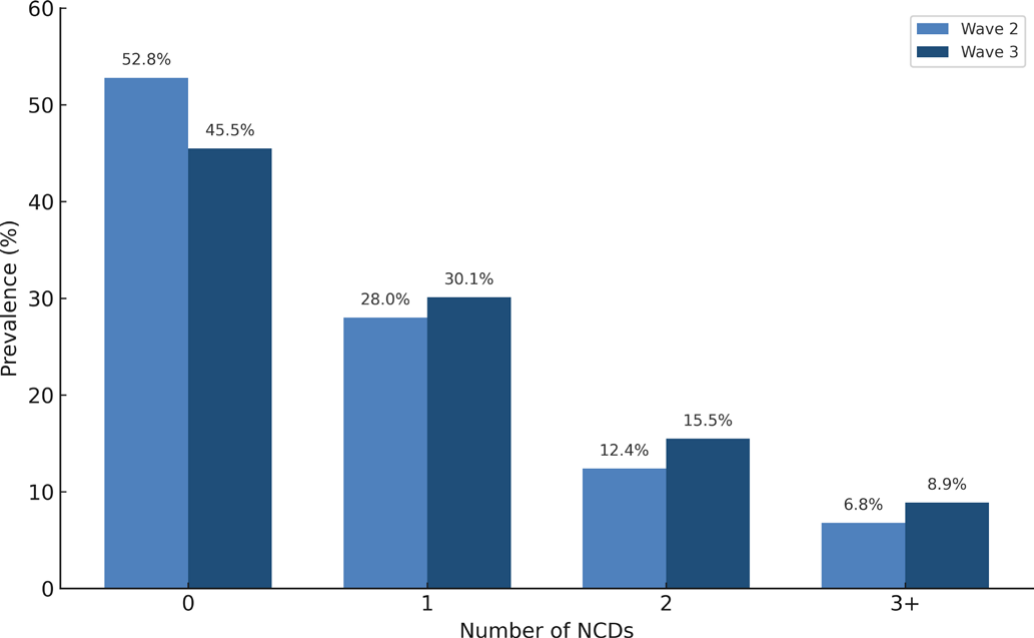
Prevalence by number of disease count. Proportion of adults aged ≥18 years with 0, 1, 2 or ≥3 self-reported chronic conditions in SAGE India Waves 2 (2015) and 3 (2019–2020). NCDs, non-communicable diseases; SAGE, Study on global AGEing and adult health.

Figure 1 shows the prevalence by number of disease count in both waves. In Wave 2, 52.8% of participants reported no chronic conditions, 28.0% had one condition, 12.4% had two and 6.8% had three or more NCDs. In Wave 3, 45.5% of participants reported no chronic conditions, while 30.1% had one, 15.5% had two and 8.9% had three or more NCDs. These distributions indicate a progressive shift toward a higher number of coexisting conditions across waves.

### Prevalence by disease combinations (dyads) stratified by age

**Figure 2:**
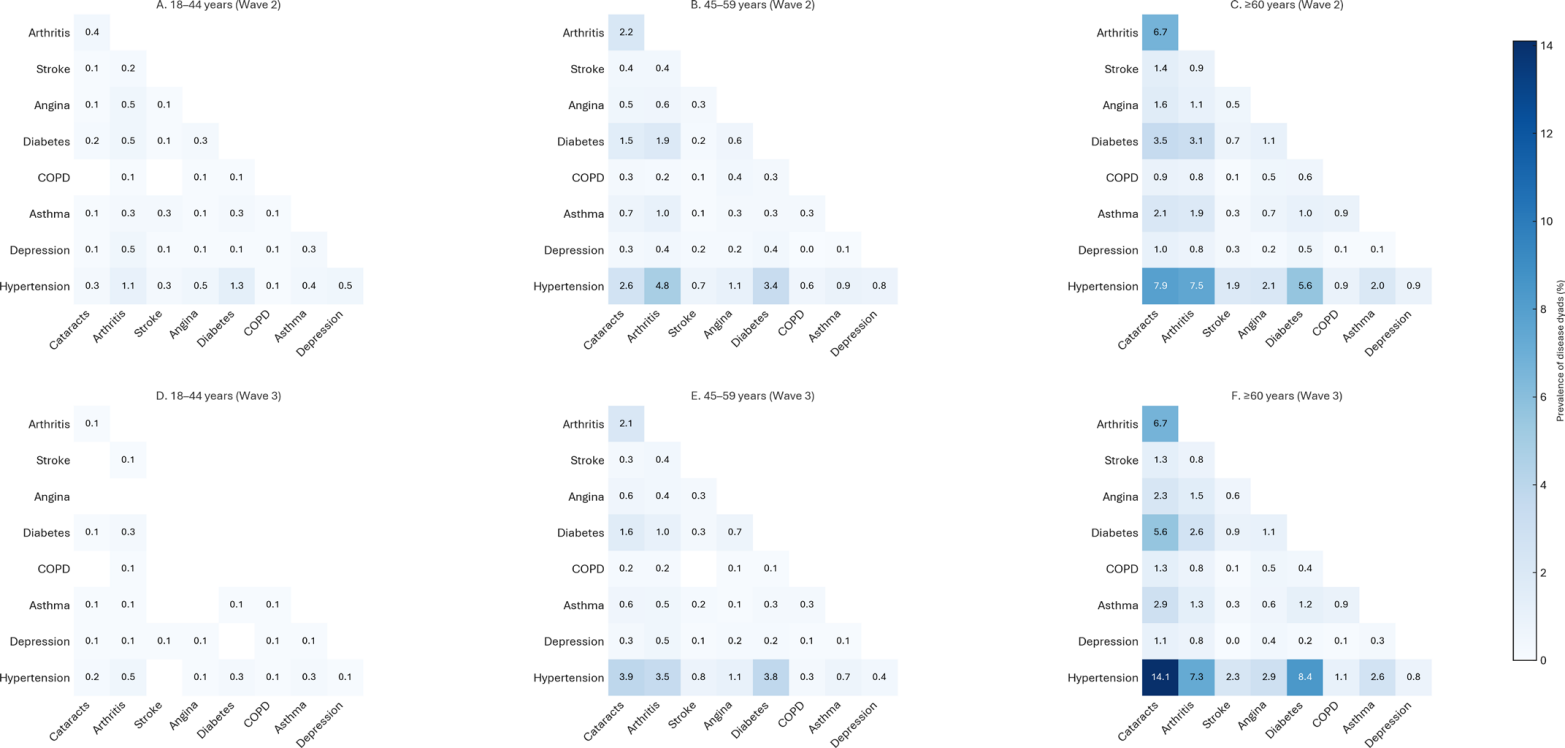
Prevalence of disease combinations by age group. Heatmap showing prevalence (%) of disease combinations among adults aged 18–44 years, 45–59 years and ≥60 years. Darker shading denotes higher prevalence. COPD, chronic obstructive pulmonary disease.

The prevalence of specific disease combinations (dyads) stratified by age group is shown in figure 2. In Wave 2, among adults aged ≥60 years, the most prevalent dyads were hypertension and cataracts (7.9%), hypertension and arthritis (7.5%), and arthritis and cataracts (6.7%). In Wave 3, hypertension and cataracts became even more common among older adults (14.1%), followed by hypertension and diabetes (8.4%) and hypertension and arthritis (7.3%). Younger age groups showed consistently lower prevalence across all disease combinations, with most dyads affecting fewer than 1% of adults aged 18–44 years in both waves.

### Quality of life score by number of disease count

**Figure 3:**
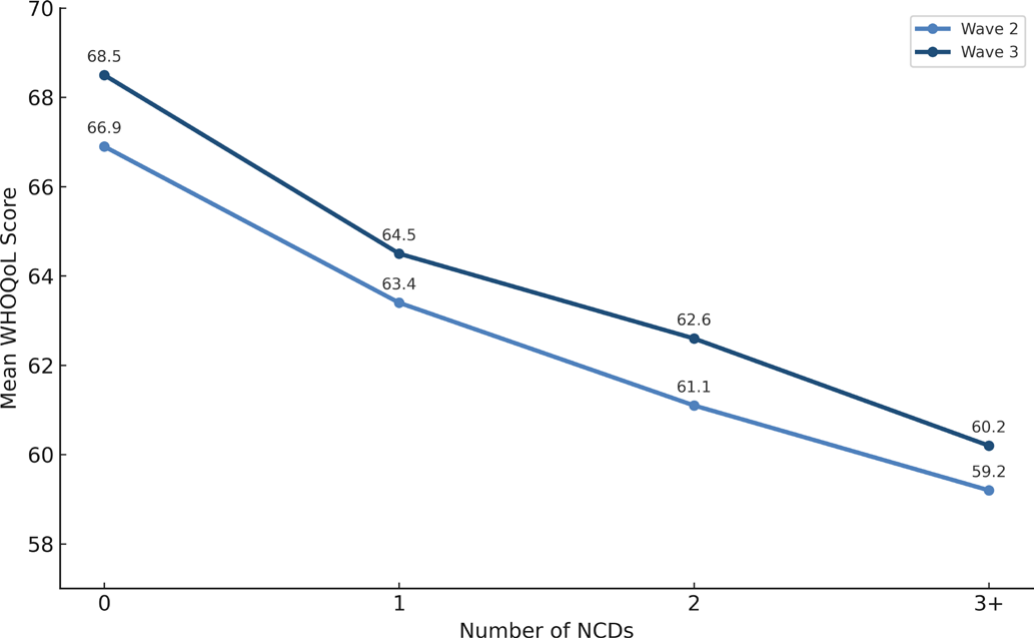
Mean WHOQoL scores by number of disease count. Mean WHOQoL-8 scores (0–100) for adults aged ≥18 years in SAGE India Waves 2 and 3, stratified by number of chronic disease count (0, 1, 2 and ≥3). NCDs, non-communicable diseases; SAGE, Study on global AGEing and adult health; WHOQoL-8, 8-item WHO Quality of Life.

Mean WHOQoL scores decreased consistently with increasing number of chronic conditions (figure 3). In Wave 2, mean quality-of-life scores were 66.86 (SD 14.93) among individuals with no conditions, 63.40 (SD 14.67) with one, 61.05 (SD 15.57) with two and 59.23 (SD 15.84) with three or more conditions. In Wave 3, the same pattern persisted, with mean scores of 68.45, 64.49, 62.59 and 60.23, respectively. Online supplemental table S2 presents mean quality-of-life scores by background characteristics.

### Quality of life by disease combinations (dyads)

**Figure 4:**
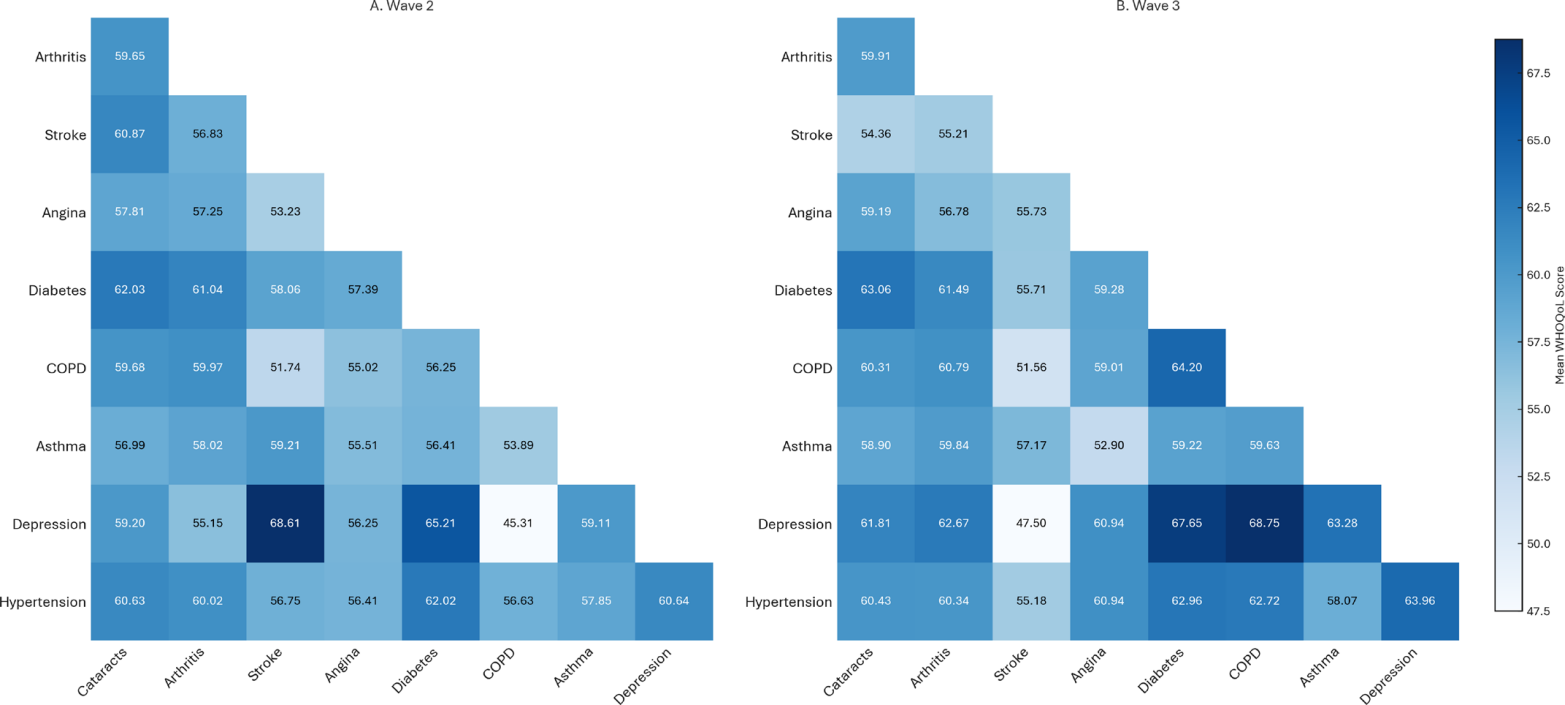
Mean WHOQoL-8 scores for disease combinations. Mean WHOQoL-8 scores for disease combinations in Wave 2 and Wave 3. Higher scores indicate better quality of life. COPD, chronic obstructive pulmonary disease; WHOQoL-8, 8-item WHO Quality of Life.

Disease-specific quality-of-life analysis showed that certain combinations were associated with particularly low WHOQoL scores (figure 4). In Wave 2, the depression–chronic obstructive pulmonary disease (COPD) dyad had the lowest mean score (45.31). In Wave 3, the lowest scores were observed for the depression–stroke combination (47.50).

### Quality of life score by domains and number of disease count

A statistically significant, progressive decline across nearly all WHOQoL-8 domains with increasing number of chronic conditions in both waves (online supplemental file 1); all p<0.001), except for the ‘Money’ domain, which was not significantly associated with disease count (p=0.271 in Wave 2; p=0.207 in Wave 3). In Wave 2, the greatest reductions were observed in ‘Overall Health’ (–15.0 points) and ‘Energy Levels’ (–11.1), followed by ‘Ability to Perform Daily Activities’ (–11.0) and ‘Self-esteem’ (–10.6). Smaller yet significant declines were noted in ‘Overall Quality of Life’ (–6.2), ‘Personal Relationships’ (–5.7) and ‘Living Conditions’ (–2.9). In Wave 3, the same pattern persisted, with pronounced declines in ‘Energy Levels’ (–15.4) and ‘Overall Health’ (–13.8), and moderate reductions in ‘Ability to Perform Daily Activities’ (–10.9), ‘Self-esteem’ (–8.0), ‘Overall Quality of Life’ (–7.0), ‘Personal Relationships’ (–6.4) and ‘Living Conditions’ (–2.3).

### Healthcare utilisation by number of disease count

**Figure 5:**
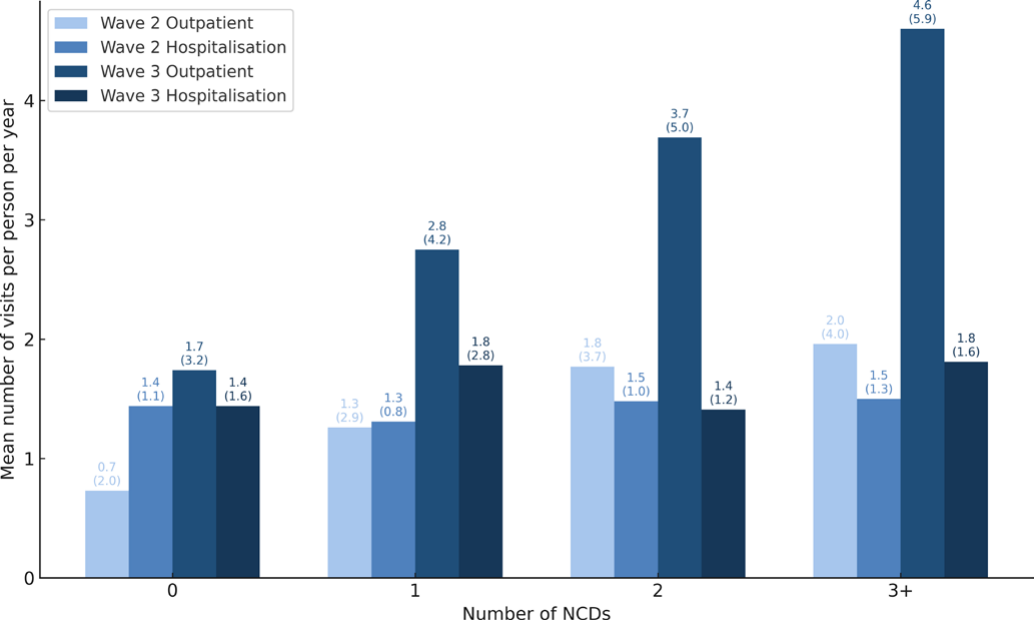
Healthcare utilisation by number of disease counts. Mean number of outpatient visits and hospitalisations in the previous 12 months among adults aged ≥18 years in Waves 2 and 3, stratified by number of chronic disease count (0, 1, 2 and ≥3). NCDs, non-communicable diseases.

Healthcare utilisation increased with the number of disease conditions in both waves (figure 5). In Wave 2, the mean number of outpatient visits per person in the preceding 12 months rose from 0.73 (SD 1.97) among individuals with no long-term conditions to 1.96 (SD 4.04) among those with three or more conditions. The mean number of hospitalisations per person over the same period remained relatively stable, ranging from 1.31 (SD 0.80) among those with one condition to 1.50 (SD 1.30) among those with three or more.

In Wave 3, a stronger gradient was observed. Mean outpatient visits increased from 1.74 (SD 3.21) among individuals with no long-term conditions to 4.60 (SD 5.94) among those with three or more. Mean hospitalisations also showed greater variability, particularly among participants with two conditions (mean 1.41, SD 1.18), but overall remained higher among those with MLTC. Online supplemental table S2 presents mean healthcare utilisation (outpatient visits and hospitalisations) by background characteristics.

### Healthcare utilisation by disease combinations (dyads)

**Figure 6:**
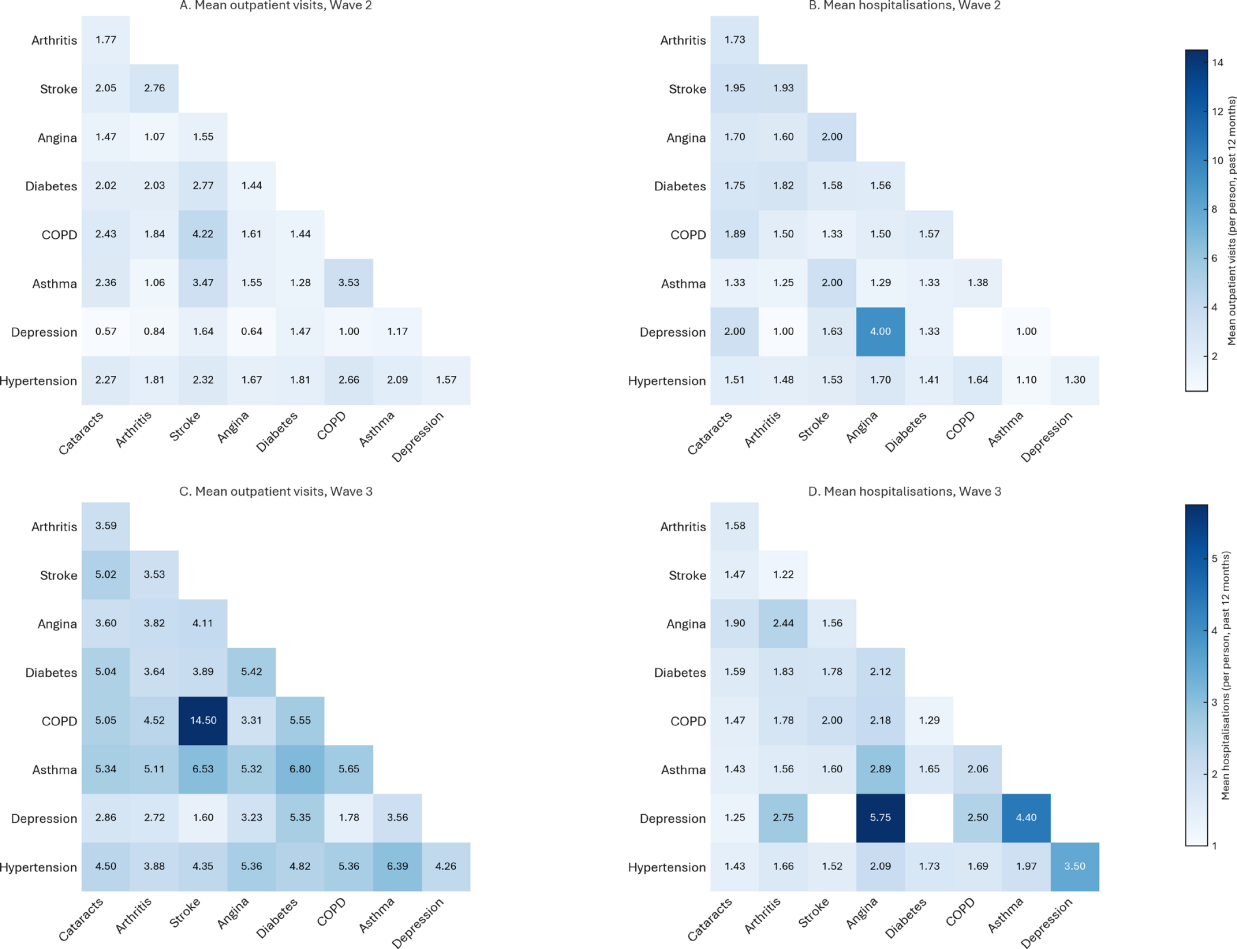
Healthcare utilisation by disease combinations. Mean outpatient visits and hospital admissions in the past 12 months by disease combinations, separately for Wave 2 and Wave 3. COPD, chronic obstructive pulmonary disease.

There was notable heterogeneity in healthcare utilisation across disease combinations (figure 6). In Wave 2, the COPD–stroke dyad was associated with the highest mean number of outpatient visits per person in the past 12 months (4.22). The depression–angina combination showed the highest mean number of hospitalisations (4.00 per person per year), surpassing all other dyads. In Wave 3, outpatient visits increased substantially for most combinations, with the COPD–stroke dyad again showing the highest mean number (14.50). The depression–angina combination continued to record the highest mean hospitalisations (5.75 per person per year), indicating persistently greater service use among these groups.

### Association between quality of life, healthcare utilisation and MLTC

The regression analysis showed a consistent dose–response relationship between the number of chronic conditions and both reduced quality of life and increased healthcare utilisation across Wave 2 (table 2) and Wave 3 (table 3). In Wave 2 (table 2), compared with participants without long-term conditions, those with one, two and three or more conditions experienced progressively greater declines in WHOQoL scores by –2.58 (95% CI –3.28 to –1.88), –5.07 (95% CI –6.02 to –4.11) and –6.69 (95% CI –7.93 to –5.44) points, respectively (all p<0.001). The number of outpatient visits increased by 0.52 (95% CI 0.47 to 0.57) for one condition, 0.86 (95% CI 0.80 to 0.92) for two and 0.96 (95% CI 0.89 to 1.04) for three or more. Hospitalisations rose incrementally from 0.47 (95% CI 0.30 to 0.64) for one condition to 1.49 (95% CI 1.29 to 1.69) for three or more (all p<0.001).

**Table 2:** Association between quality of life, healthcare utilisation and number of MLTC (Wave 2)

Characteristics	QOL score (Tobit regression; coeff. with 95% CI)	Outpatient visits (Poisson regression; coeff. with 95% CI)	Hospitalisations (Poisson regression; coeff. with 95% CI)
Number of MLTC (Ref: 0)			
1 NCD	−2.575*** (−3.275 to −1.875)	0.523*** (0.471 to 0.574)	0.469*** (0.297 to 0.640)
2 NCDs	−5.068*** (−6.021 to −4.114)	0.860*** (0.800 to 0.920)	0.967*** (0.774 to 1.159)
3+ NCDs	−6.687*** (−7.931 to −5.443)	0.963*** (0.891 to 1.035)	1.487*** (1.285 to 1.689)
Sex (Ref: female)			
Male	1.799*** (1.066 to 2.533)	−0.174*** (−0.227 to −0.121)	0.263** (0.099 to 0.428)
Age group (Ref: 18–44 years)			
45–59 years	−3.258*** (−4.215 to −2.301)	−0.002 (−0.073 to 0.070)	−0.146 (−0.376 to 0.084)
60+ years	−4.420*** (−5.425 to −3.415)	0.050 (−0.025 to 0.124)	−0.061 (−0.297 to 0.174)
Marital status (Ref: never married)			
Currently married/cohabiting	−1.188 (−2.640 to 0.264)	0.185** (0.062 to 0.308)	0.484* (0.065 to 0.902)
Separated/divorced	−3.439 (−7.795 to 0.917)	0.277 (−0.013 to 0.568)	0.672 (−0.236 to 1.580)
Widowed	−4.558*** (−6.219 to −2.897)	0.218** (0.084 to 0.352)	0.144 (−0.316 to 0.605)
Ethnic group (Ref: schedule tribe)			
Schedule caste	−0.349 (−1.595 to 0.897)	0.363*** (0.267 to 0.458)	−0.210 (−0.480 to 0.060)
Other backward caste	1.687** (0.540 to 2.833)	0.119* (0.027 to 0.210)	−0.167 (−0.412 to 0.079)
None of the above	−0.283 (−1.588 to 1.022)	0.105* (0.002 to 0.207)	−0.224 (−0.509 to 0.061)
Others	−2.137** (−3.487 to −0.786)	0.296*** (0.195 to 0.397)	−0.250 (−0.544 to 0.043)
Religion (Ref: Hinduism)			
Islam	−3.862*** (−4.783 to −2.942)	0.192*** (0.131 to 0.253)	−0.000 (−0.205 to 0.205)
Others	−1.875* (−3.397 to −0.353)	−0.425*** (−0.555 to −0.295)	0.329* (0.043 to 0.615)
Years of schooling (Ref: no formal schooling)			
1–5 years	−1.049* (−1.913 to −0.184)	−0.055 (−0.115 to 0.005)	0.062 (−0.123 to 0.246)
6–9 years	1.423** (0.497 to 2.349)	0.042 (−0.021 to 0.105)	0.068 (−0.130 to 0.267)
10+ years	2.812*** (1.864 to 3.760)	−0.180*** (−0.249 to −0.110)	−0.183 (−0.397 to 0.032)
Wealth quintiles (Ref: lowest)			
Second	2.137*** (1.190 to 3.085)	−0.115** (−0.185 to −0.045)	−0.095 (−0.313 to 0.122)
Middle	3.289*** (2.331 to 4.246)	0.204*** (0.138 to 0.270)	0.007 (−0.207 to 0.222)
Fourth	6.648*** (5.679 to 7.618)	−0.015 (−0.085 to 0.055)	−0.024 (−0.243 to 0.194)
Highest	10.911*** (9.883 to 11.939)	0.034 (−0.040 to 0.108)	−0.073 (−0.305 to 0.159)
Health insurance (Ref: no)			
Yes	0.033 (−0.955 to 1.021)	0.464*** (0.407 to 0.521)	0.163 (−0.038 to 0.364)
Residence (Ref: rural)			
Urban	−0.315 (−1.105 to 0.474)	0.179*** (0.126 to 0.233)	0.062 (−0.110 to 0.234)
Body mass index (Ref: normal weight)			
Underweight	−2.547*** (−3.251 to −1.842)	0.191*** (0.142 to 0.240)	0.253** (0.096 to 0.410)
Overweight	−0.410 (−1.286 to 0.465)	0.070* (0.010 to 0.130)	0.236* (0.051 to 0.420)
Obese	0.117 (−1.469 to 1.704)	−0.079 (−0.191 to 0.033)	0.020 (−0.326 to 0.365)
Physical activity (Ref: low)			
Moderate	0.979 (−0.112 to 2.070)	−0.163*** (−0.242 to −0.085)	−0.225* (−0.443 to −0.007)
High	0.562 (−0.569 to 1.692)	0.389*** (0.311 to 0.466)	−0.156 (−0.383 to 0.072)
Tobacco use (Ref: never)			
Current	−0.987** (−1.722 to −0.252)	0.238*** (0.188 to 0.288)	−0.090 (−0.252 to 0.073)
Past	−1.768* (−3.362 to −0.174)	0.510*** (0.420 to 0.600)	0.382** (0.117 to 0.647)
Alcohol consumption (Ref: person who abstains from alcohol for life)			
Person who does not drink heavily	−2.717*** (−3.982 to −1.452)	0.152*** (0.074 to 0.231)	0.217 (−0.022 to 0.455)
Person who infrequently drinks alcohol heavily	−1.043 (−4.353 to 2.267)	−0.276* (−0.536 to −0.015)	−1.013 (−2.152 to 0.125)
Person who frequently drinks alcohol heavily	−0.344 (−2.642 to 1.954)	−0.239** (−0.413 to −0.065)	0.489* (0.110 to 0.869)
Constant	66.214*** (64.136 to 68.292)	−0.949*** (−1.117 to −0.781)	−2.901*** (−3.423 to −2.378)
	n=8329	n=8366	n=8366
	Likelihood ratio χ² (34) = 1719.29, p<0.001	Likelihood ratio χ² (34) = 3075.27, p<0.001	Likelihood ratio χ² (34) = 360.80, p<0.001

*p<0.05; **p<0.01; ***p<0.001.

Model adjusted for all variables shown. Quality of life estimated using Tobit regression; outpatient visits and hospitalisations estimated using Poisson regression. Positive coefficients indicate higher quality of life scores and higher counts of outpatient visits or hospitalisations. Likelihood ratio χ² tests overall model fit against an intercept-only model.

MLTC, multiple long-term condition; NCD, non-communicable disease; QOL, Quality of Life.

**Table 3:** Association between quality of life, healthcare utilisation and number of MLTC (Wave 3)

Characteristics	QOL score (Tobit regression; coeff. with 95% CI)	Outpatient visits (Poisson regression; coeff. with 95% CI)	Hospitalisations (Poisson regression; coeff. with 95% CI)
Number of long-term conditions (Ref: 0)			
1 NCD	−3.308*** (−3.998 to −2.618)	0.408*** (0.371 to 0.445)	0.886*** (0.732 to 1.040)
2 NCDs	−5.629*** (−6.506 to −4.752)	0.663*** (0.621 to 0.705)	0.920*** (0.739 to 1.101)
3+NCDs	−7.948*** (−9.052 to −6.844)	0.908*** (0.861 to 0.955)	1.564*** (1.383 to 1.745)
Sex (Ref: female)			
Male	2.583*** (1.839 to 3.326)	0.058** (0.020 to 0.095)	0.135 (−0.011 to 0.281)
Age Group (Ref: 18–44 years)			
45–59 years	−3.362*** (−4.440 to −2.284)	−0.013 (−0.075 to 0.049)	−0.096 (−0.348 to 0.155)
60+ years	−4.731*** (−5.847 to −3.615)	−0.005 (−0.069 to 0.059)	0.024 (−0.230 to 0.278)
Marital status (Ref: never married)			
Currently married/cohabiting	1.523 (−0.216 to 3.263)	0.370*** (0.246 to 0.494)	0.221 (−0.227 to 0.669)
Separated/divorced	−2.443 (−6.608 to 1.721)	−0.035 (−0.302 to 0.232)	−0.662 (−1.880 to 0.556)
Widowed	−0.914 (−2.810 to 0.982)	0.392*** (0.263 to 0.521)	0.044 (−0.428 to 0.516)
Ethnic group (Ref: schedule tribe)			
Schedule caste	−0.574 (−1.821 to 0.674)	0.135*** (0.067 to 0.202)	0.258 (−0.027 to 0.544)
Other backward caste	1.158 (−0.012 to 2.329)	0.190*** (0.127 to 0.253)	0.413** (0.148 to 0.679)
None of the above	−0.240 (−1.435 to 0.955)	0.092** (0.027 to 0.157)	0.369** (0.098 to 0.640)
Others	2.431* (0.551 to 4.310)	−0.087 (−0.194 to 0.020)	0.161 (−0.242 to 0.564)
Religion (Ref: Hinduism)			
Islam	−3.657*** (−4.597 to −2.718)	0.233*** (0.190 to 0.275)	−0.035 (−0.218 to 0.148)
Others	−4.824*** (−6.050 to −3.598)	−0.317*** (−0.391 to −0.243)	0.172 (−0.055 to 0.399)
Years of schooling (Ref: no formal schooling)			
1–5 years	−0.673 (−1.475 to 0.129)	−0.138*** (−0.177 to −0.099)	0.281*** (0.131 to 0.430)
6–9 years	0.722 (−0.191 to 1.634)	−0.270*** (−0.315 to −0.224)	0.124 (−0.053 to 0.300)
10+ years	3.500*** (2.547 to 4.453)	−0.426*** (−0.475 to −0.377)	−0.058 (−0.249 to 0.133)
Wealth quintiles (Ref: lowest)			
Second	2.350*** (1.425 to 3.275)	0.117*** (0.067 to 0.167)	0.217* (0.027 to 0.406)
Middle	3.578*** (2.646 to 4.510)	0.203*** (0.154 to 0.252)	0.157 (−0.034 to 0.349)
Fourth	5.796*** (4.842 to 6.750)	0.286*** (0.236 to 0.335)	0.159 (−0.033 to 0.352)
Highest	8.781*** (7.756 to 9.807)	0.462*** (0.411 to 0.514)	−0.322** (−0.539 to −0.104)
Health insurance (Ref: no)			
Yes	−1.758*** (−2.402 to −1.115)	0.230*** (0.199 to 0.260)	−0.052 (−0.179 to 0.075)
Residence (Ref: rural)			
Urban	−0.103 (−0.903 to 0.697)	−0.115*** (−0.155 to −0.075)	0.139 (−0.008 to 0.286)
Body mass index (Ref: normal weight)			
Underweight	−2.787*** (−3.549 to −2.025)	0.104*** (0.066 to 0.142)	−0.088 (−0.245 to 0.069)
Overweight	1.692*** (0.916 to 2.469)	0.108*** (0.071 to 0.146)	0.038 (−0.107 to 0.182)
Obese	1.020 (−0.258 to 2.297)	0.015 (−0.046 to 0.076)	−0.294* (−0.558 to −0.031)
Physical activity (Ref: low)			
Moderate	0.296 (−0.910 to 1.501)	−0.162*** (−0.218 to −0.106)	0.172 (−0.060 to 0.403)
High	0.891 (−0.329 to 2.110)	−0.017 (−0.073 to 0.040)	−0.029 (−0.267 to 0.209)
Tobacco use (Ref: never)			
Current	−1.236*** (−1.939 to −0.533)	−0.045* (−0.081 to −0.010)	−0.297*** (−0.437 to −0.156)
Past	−3.121*** (−4.401 to −1.841)	−0.023 (−0.085 to 0.039)	−0.490*** (−0.753 to −0.226)
Alcohol consumption (Ref: person who abstains from alcohol for life)			
Person who does not drink heavily	−0.667 (−1.875 to 0.541)	0.070* (0.011 to 0.129)	0.534*** (0.333 to 0.734)
Person who infrequently drinks alcohol heavily	−0.186 (−5.580 to 5.208)	−0.015 (−0.289 to 0.259)	0.082 (−0.908 to 1.071)
Person who frequently drinks alcohol heavily	0.366 (−2.333 to 3.065)	0.132* (0.000 to 0.263)	−0.024 (−0.580 to 0.532)
Constant	66.217*** (63.841 to 68.593)	−0.006 (−0.156 to 0.143)	−3.163*** (−3.732 to −2.594)
	n=7461	n=7463	n=7463
	Likelihood ratio χ² (34) = 1746.33, p<0.001	Likelihood ratio χ² (34) = 3884.84 p<0.001	Likelihood ratio χ² (34) = 524.224, p<0.001

*p<0.05; **p<0.01; ***p<0.001.

Model adjusted for all variables shown. Quality of life estimated using Tobit regression; outpatient visits and hospitalisations estimated using Poisson regression. Positive coefficients indicate higher quality of life scores and higher counts of outpatient visits or hospitalisations. Likelihood ratio χ² tests overall model fit against an intercept-only model.

MLTC, multiple long-term condition; NCD, non-communicable disease; QOL, Quality of Life.

In Wave 3 (table 3), a similar pattern was observed. WHOQoL scores declined by –3.31 (95% CI –4.00 to –2.62), –5.63 (95% CI –6.51 to –4.75) and –7.95 (95% CI –9.05 to –6.84) points for one, two and three or more conditions, respectively (all p<0.001). Outpatient visits increased by 0.41 (95% CI 0.37 to 0.45) for one condition, 0.66 (95% CI 0.62 to 0.71) for two and 0.91 (95% CI 0.86 to 0.96) for three or more. Hospitalisations rose from 0.89 (95% CI 0.73 to 1.04) to 1.56 (95% CI 1.38 to 1.75) (all p<0.001). Beyond MLTC, several sociodemographic and behavioural factors including sex, wealth, education, physical activity and tobacco or alcohol use were independently associated with quality of life and healthcare utilisation in both survey waves (tables 2 and 3).

## Discussion

Using data from Waves 2 and 3 of the WHO SAGE survey, this study examined prevalence, patterns and associations of MLTC with WHOQoL and healthcare utilisation among adults in India. The findings show a higher observed prevalence of MLTC in Wave 3 compared with Wave 2, with greater concentration among older adults, women, urban residents and wealthier groups. However, as the two waves represent separate cross-sectional samples with differing age structures, these differences should be interpreted as descriptive rather than indicative of a true temporal increase. Hypertension combined with cataracts, arthritis or diabetes emerged as the most common disease dyads, while depression with COPD (Wave 2) and depression with stroke (Wave 3) were associated with the poorest quality of life. Physical and functional domains such as overall health, energy levels and daily activities were most severely affected, whereas financial satisfaction remained relatively stable. Outpatient utilisation increased with condition count, particularly for respiratory and neurological combinations, whereas hospitalisation patterns were less consistent.

The rising prevalence of MLTC aligns with systematic review reporting pooled prevalence of 20% in India^14^ and mirrors global trends in LMICs, where MLTC are increasing more rapidly than in high-income countries and at younger ages.^6–8 45^ Although lower than the global pooled prevalence of 37.2% across 54 countries,^4^ the SAGE estimates may underestimate the burden due to self-reporting, underdiagnosis, inclusion of younger adults and limited number of conditions used. The most common dyads observed were consistent with multicountry SAGE analyses and other studies, confirming the predominance of hypertension-related combinations.^29 46^

Sociodemographic and socio-economic patterns were also consistent with earlier Indian studies, showing higher prevalence among women and widowed participants.^15 47 48^ The higher burden among wealthier groups may reflect greater access to healthcare and diagnosis, contrasting with high-income settings where MLTC is concentrated in more deprived and ethnic minority populations.^14 49–52^ This highlights the importance of healthcare access in shaping observed disease patterns in India.

The dyad patterns identified in this analysis are consistent with a substantial international literature on MLTC clustering. Barnett and colleagues’ landmark Scottish study of 1.75 million patients showed that physical–mental comorbidity becomes more common as both condition count and socio-economic deprivation increase.^51^ Using exploratory factor analysis in 275 682 adult patients from Spanish primary care, Prados-Torres and colleagues identified five recurring multimorbidity patterns including cardiometabolic and depressive clusters.^53^ Cross-national analyses, including a systematic review of eight Asian studies and harmonised cohort data spanning LMICs and high-income countries, have consistently identified cardiovascular–metabolic and mental health as the dominant clusters, alongside musculoskeletal and respiratory groupings.^54–56^ Despite heterogeneity in analytical methods factor analysis, hierarchical clustering and latent class analysis, three broad groupings have emerged consistently across settings: cardiovascular–metabolic, mental health and musculoskeletal or respiratory conditions.^54^ A European study found three broadly consistent patterns in both Spain and the Netherlands and showed that the cardiometabolic pattern increased in complexity with age.^57^ The present findings fit this picture closely. The hypertension-dominant dyads reflect the cardiometabolic cluster; the depression–COPD and depression–stroke combinations represent the mental–physical cluster in an Indian epidemiological context.

The particularly low WHOQoL-8 scores for depression-inclusive dyads (45.3 for depression–COPD; 47.5 for depression–stroke) reflect the distinct functional burden these combinations impose. Unlike largely asymptomatic conditions such as hypertension or early-stage diabetes, depression, COPD and stroke each directly impair the domains the WHOQoL-8 measures: depression through reduced energy, self-esteem and perceived functioning; COPD through breathlessness and activity limitation; stroke through acute multi-domain disability. Several WHOQoL-8 domains, including energy, self-esteem and ability to perform daily activities, closely resemble core depressive symptom domains; prior work using a related instrument has noted that this content overlap may amplify depression–QoL associations.^42^ Schrock and colleagues, using WHO SAGE Wave 1 data from adults in six middle-income countries, found that greater cumulative chronic morbidity was associated with higher subjective fatigue, with a clear dose-response pattern.^58^ This is consistent with the energy and functional domain declines observed here.

A clear dose-response relationship was evident between disease count and WHOQoL-8 scores, with individuals reporting three or more conditions experiencing the largest adjusted reduction in quality of life. This pattern aligns with prior evidence reporting cumulative decrements in quality of life with each additional disease.^27 59 60^ The relationship is not, however, a direct one. A mediation analysis using WHO SAGE data from six LMICs found that mobility limitations explained 47.9% of the association between physical multimorbidity and quality of life, with pain/discomfort (43.5%) and sleep/energy (35.0%) also mediating this relationship.^61^ Longitudinal and mediation studies indicate that the association between MLTC (or higher disease counts) and poorer quality of life is partly explained by intermediate pathways, particularly functional limitations and health-system or socio-economic factors.^24 62 63^ The disproportionate declines observed in the physical, energy and self-esteem domains of WHOQoL-8, set against the relative stability of the financial satisfaction domain, support this interpretation: people may experience reduced quality of life because chronic conditions constrain what they can do and how they perceive themselves, rather than as a direct consequence of disease count alone. Causal claims warrant caution, given the cross-sectional design of each wave and the likelihood that observed associations reflect mediated rather than direct effects.

Although the analytical sample spans adults aged 18 years and above, the multimorbidity burden was heavily concentrated in later life: prevalence in Wave 3 reached 32.3% among adults aged 60 years and older, compared with 3.2% among those aged 18–44 years. The financial domain of WHOQoL-8 remained stable across disease counts in both waves, while the physical and functional domains overall health, energy levels and ability to perform daily activities fell most sharply. This pattern suggests that the lived experience of multimorbidity is shaped primarily through functional and physical pathways rather than through perceived financial strain, at least within the age range and socio-economic context of this sample. Qualitative studies have highlighted how multimorbidity generates a multifaceted physical and functional workload, resulting in substantial burdens and disruption to everyday life.^64^ Future age-stratified analyses using longitudinal Indian data would help clarify how these functional impacts vary across the adult life course. Observed declines in physical function and energy levels, alongside stable financial satisfaction, align with the WHO’s intrinsic capacity framework, which frames healthy ageing in terms of functional ability rather than the mere absence of disease.^65^

Outpatient visits increased sharply with condition count, particularly in dyads involving COPD and stroke, reflecting excess service demand similar to trends observed in high-income settings.^66 67^ Hospitalisation patterns, although less consistent, showed greater likelihood of hospitalisation among individuals with more than two chronic conditions, consistent with previous findings and possibly reflecting fragmented care or access barriers.^66 68^ Insurance was also positively associated with outpatient visits across both waves, suggesting that financial protection mechanisms may facilitate service use among individuals with multiple conditions. However, insurance did not consistently predict hospitalisations, which may depend more on illness severity, referral pathways and availability of inpatient services.

Sociodemographic and behavioural factors associated with quality of life and healthcare utilisation highlight important areas for intervention. The more favourable outcomes observed among men, individuals with higher educational attainment, those in higher wealth quintiles and those who are physically active underscore persistent socio-economic and gender-based health inequities in India. These findings align with global evidence showing that MLTC burden is concentrated among socio-economically disadvantaged groups, creating a ‘double burden’ where those with the greatest disease burden also have the least resources to manage it effectively.^69–71^ The association between health insurance and higher outpatient use but lower quality of life in Wave 3 may reflect adverse selection, with sicker individuals more likely to obtain coverage.

The operational definition of MLTC used here, defined as two or more chronic conditions, follows widespread convention but requires critical reflection. The ≥2 threshold treats hypertension co-occurring with cataracts as analytically equivalent to depression co-occurring with stroke, despite the very different consequences for daily life evident in our domain-specific results, where depression-COPD and depression-stroke dyads were associated with the lowest WHOQoL-8 scores (45.3 and 47.5, respectively). Ho and colleagues’ Delphi consensus confirmed the ≥2 threshold but emphasised the need for greater standardisation.^72^ The study reached agreement on 24 conditions to always include in multimorbidity measures and a further 35 to usually include. Simple counts were preferred for prevalence and clustering, weighted measures for risk adjustment and outcome prediction. The ≥2 threshold may also carry different meanings across the age range, with two co-occurring conditions in a person aged 30 years representing a clinically distinct phenomenon from the same count in a person aged 70 years. Our use of nine conditions available from the SAGE dataset excludes several common burdens such as cardiovascular disease beyond angina, cancer, dementia and chronic kidney disease, so estimates here probably understate true MLTC prevalence. Sensitivity analyses using weighted indices were not possible because the dataset does not capture severity, and the limited condition set restricts the analytical value of alternative count-based thresholds. Future work using linked electronic health record data or expanded survey instruments would allow a more detailed examination of how threshold and condition choice shape estimates of burden.

### Strengths and limitations

Strengths include the use of two large, nationally representative SAGE surveys, standardised WHOQoL-8 assessment and regression modelling adjusting for a wide range of sociodemographic and behavioural confounders. The study also advances understanding by identifying common MLTC combinations affecting quality of life, examining outcomes by both disease count and dyads across QoL domains, incorporating behavioural factors and comparing two survey waves, one of them recent. Limitations include reliance on self-reported diagnoses and healthcare use, which may introduce misclassification and recall bias, and data restricted to six states, which may not fully reflect regional and cultural diversity. The data also had information on nine specific conditions, in contrast to suggestions that a larger uniform number of conditions should be used for MLTC research.^72^ The cross-sectional analysis of each wave limits causal inference, while the brief WHOQoL-8 may not capture disease-specific aspects of QoL. Formal mediation analysis was not undertaken, as several WHOQoL-8 items that could serve as candidate mediators are themselves components of the composite outcome. Differences in MLTC prevalence between waves should therefore be interpreted with caution, as the two samples varied in age and other characteristics and the data do not track the same individuals over time. Despite adjusting for multiple confounders, unmeasured factors such as diet, social support and healthcare quality were not assessed. Finally, healthcare utilisation measures were limited to inpatient and outpatient visits, excluding medication use and informal care.

### Implications on policy, practice and research

This study underscores the need for India’s health system to move beyond single-disease models and prioritise high-burden MLTC clusters, particularly depression–COPD, depression–stroke and COPD–stroke, which are strongly associated with poorer quality of life and higher outpatient use. Policy should direct resources towards these combinations, strengthen multidisciplinary care and reduce socio-economic inequities affecting older adults, women, urban residents and disadvantaged groups. For practice, providers must recognise the cumulative impact of multiple chronic conditions on functional health and service demand. Future research should prioritise longitudinal designs and intervention studies to test targeted MLTC management models. Examining mediating mechanisms will require longitudinal data with functional status, symptom burden and psychological well-being measured independently of the composite quality-of-life outcome. Cross-sectional mediation analyses using WHO SAGE data^61^ and structural equation modelling with Indian survey data^24^ have begun to examine how MLTC, functional limitations and socio-economic disadvantages relate to quality of life in older adults. Future surveys should incorporate independently measured functional and clinical assessments alongside WHOQoL-8 to make such analyses possible. Future surveys should also expand the range of conditions captured to enable more precise estimates of MLTC prevalence and burden.

## Conclusions

The rising prevalence of MLTC in India disproportionately affects older adults, women, urban residents and wealthier groups. Hypertension frequently co-occurred with cataracts, arthritis or diabetes, while MLTC overall was associated with poorer quality of life, particularly in physical and functional domains and greater outpatient use. These findings highlight how specific disease combinations shape health outcomes and service demand, providing timely evidence to guide India’s preparedness for the growing MLTC burden.

## Supplementary material

10.1136/bmjopen-2026-116399 online supplemental file 1

## Data Availability

Data may be obtained from a third party and are not publicly available.
