# Supplementary material for: Multiple long-term conditions and their association with quality of life and healthcare utilisation among adults in India: a cross-sectional analysis of WHO SAGE Waves 2 and 3
Source: BMJ Open. 2026 Aug 11;16(8):e116399. doi: 10.1136/bmjopen-2026-116399 (PMC13475192; doi:10.1136/bmjopen-2026-116399)
Supplement: Supplementary file 1 [file bmjopen-16-8-s001.pdf]

Supplementary Files

Table S1: Detailed definitions of covariates

Variable	Category	Definition
Body mass index (BMI)	Underweight	BMI calculated as weight in kilograms divided by height in meters squared (kg/m^2) classified using WHO cut-off points<br><br>BMI less than 18.5 kg/m^2
	Normal weight	BMI between 18.5 to 24.99 kg/m^2
	Overweight	BMI between 25.0 to 29.99 kg/m^2
	Obese	BMI 30 kg/m^2 or greater
Physical activity (IPAQ protocol)	High	Vigorous-intensity activity on at least 3 days, accumulating ≥1500 MET-min/week, OR 7 or more days of any combination of walking, moderate- or vigorous-intensity activities accumulating ≥3000 MET-min/week
	Moderate	Three or more days of vigorous-intensity activity of at least 20 minutes per day, OR five or more days of moderate-intensity activity and/or walking of at least 30 minutes per day, OR five or more days of any combination of walking, moderate- or vigorous-intensity activities accumulating ≥600 MET-min/week
	Low	Does not meet criteria for either 'Moderate' or 'High' categories
Tobacco use	Never	Respondent has never used any form of tobacco (smoked or smokeless) in their lifetime
	Current	Respondent currently uses any form of tobacco (smoked or smokeless), either daily or less than daily
	Past	Respondent previously used any form of tobacco (smoked or smokeless) but is not a current use
Alcohol consumption	Lifetime abstainer	Never consumed alcoholic beverages in their lifetime
	Non-heavy drinker	No alcohol in the past year, OR less than once a month, OR one to three days per month, AND fewer than five standard drinks in the past seven days
	Infrequent heavy drinker	Consumed alcohol 1–4 days per week with fewer than five standard drinks in the past seven days
	Frequent heavy drinker	Consumed alcohol on five or more days per week with five or more standard drinks in the past seven days

Table S2 Mean quality of life score and healthcare utilisation (outpatient visits and hospitalisations) by background characteristics

	Wave 2			Wave 3		
	Mean WHOQoL Score (SD)*	Mean outpatient visits (SD)	Mean hospitalisations (SD)	Mean WHOQoL Score (SD)*	Mean outpatient visits (SD)	Mean hospitalisations (SD)
**Sex**						
Female	63.51 (15.12)	1.12 (2.58)	1.34 (0.84)	64.11 (13.81)	2.71 (4.32)	1.60 (2.17)
Male	65.99 (15.23)	1.06 (2.86)	1.50 (1.23)	67.47 (14.28)	2.47 (4.13)	1.62 (1.75)
**Age in Years**						
18-44 years	70.18 (14.74)	0.84 (2.14)	1.29 (0.63)	72.64 (12.99)	1.68 (3.11)	1.44 (1.13)
45-59 years	65.41 (14.36)	1.05 (2.61)	1.40 (0.96)	67.02 (13.35)	2.43 (4.08)	1.61 (1.71)
60+ years	62.02 (15.45)	1.21 (2.95)	1.46 (1.19)	63.38 (14.24)	2.89 (4.49)	1.63 (2.17)
**Marital Status**						
Never married	71.40 (15.74)	0.66 (1.42)	1.21 (0.41)	71.78 (14.96)	1.16 (2.09)	2.00 (1.90)
Currently married/cohabiting	65.64 (14.71)	1.08 (2.68)	1.47 (1.14)	66.82 (13.56)	2.58 (4.20)	1.64 (2.12)
Separated/divorced	61.73 (17.17)	1.22 (2.10)	1.17 (0.41)	62.93 (17.31)	1.64 (1.97)	1.00 (0.00)
Widowed	59.01 (15.32)	1.23 (3.06)	1.26 (0.72)	60.65 (14.55)	2.90 (4.60)	1.51 (1.47)
**Ethnic group**						
Schedule Tribe	63.23 (15.99)	0.81 (2.33)	1.69 (1.38)	63.24 (13.78)	2.10 (4.15)	1.69 (1.92)
Schedule Caste	63.03 (15.10)	1.26 (2.98)	1.43 (1.11)	63.64 (13.78)	2.46 (3.79)	1.56 (1.79)
Other Backward Caste	65.91 (14.86)	1.05 (2.65)	1.34 (0.97)	66.65 (13.82)	2.86 (4.56)	1.59 (1.44)
None of the Above	64.83 (14.93)	0.96 (2.49)	1.50 (1.07)	65.88 (14.53)	2.57 (4.16)	1.67 (2.57)
Others	62.98 (16.02)	1.33 (2.98)	1.46 (1.02)	67.20 (13.78)	2.04 (3.63)	1.29 (0.74)
**Religion**						
Hinduism	65.27 (15.15)	1.08 (2.72)	1.41 (1.00)	66.41 (14.07)	2.58 (4.17)	1.60 (1.74)
Islam	60.86 (15.01)	1.23 (2.88)	1.39 (1.20)	62.11 (13.91)	3.27 (5.02)	1.50 (2.91)
Others	63.16 (15.42)	0.78 (1.82)	1.71 (1.49)	61.09 (13.51)	1.62 (3.21)	2.07 (2.73)
**Years of schooling**						
No formal schooling	61.92 (15.02)	1.09 (2.87)	1.37 (1.04)	62.54 (14.09)	2.95 (4.59)	1.44 (1.37)
1-5 years	62.45 (15.21)	1.18 (2.47)	1.49 (1.13)	63.53 (13.24)	2.62 (4.13)	1.87 (3.06)
6-9 years	66.71 (14.31)	1.23 (2.81)	1.50 (1.10)	67.39 (13.26)	2.42 (4.05)	1.76 (2.00)
10+ years	70.69 (14.33)	0.93 (2.45)	1.41 (0.96)	72.18 (13.23)	2.05 (3.68)	1.55 (1.31)

**Wealth quintiles**						
Lowest	59.07 (15.39)	1.03 (2.88)	1.61 (1.38)	60.35 (14.14)	2.08 (3.72)	1.52 (1.53)
Second	61.84 (14.03)	0.94 (2.31)	1.41 (1.16)	63.35 (13.62)	2.39 (3.83)	1.56 (2.09)
Middle	63.45 (14.57)	1.30 (3.06)	1.45 (1.12)	64.97 (13.46)	2.56 (4.40)	1.92 (3.04)
Fourth	66.53 (14.87)	1.08 (2.61)	1.34 (0.76)	67.36 (13.80)	2.80 (4.07)	1.64 (1.67)
Highest	71.20 (14.23)	1.10 (2.63)	1.36 (0.86)	71.17 (13.24)	3.09 (4.90)	1.43 (1.21)
**Health insurance**						
No	64.54 (15.11)	1.02 (2.58)	1.42 (1.06)	66.16 (13.73)	2.39 (4.35)	1.72 (2.22)
Yes	65.64 (16.15)	1.70 (3.62)	1.41 (0.98)	64.20 (15.01)	3.16 (3.86)	1.37 (1.28)
**Place of residence**						
Rural	63.97 (15.24)	1.04 (2.66)	1.41 (1.08)	64.97 (14.01)	2.61 (4.32)	1.53 (1.69)
Urban	67.17 (14.85)	1.28 (2.87)	1.44 (0.96)	68.45 (14.29)	2.56 (3.85)	1.95 (2.93)
**Body mass index**						
Underweight	61.46 (15.28)	1.19 (2.79)	1.47 (1.26)	61.36 (14.54)	2.65 (4.63)	1.54 (1.40)
Normal weight	65.86 (14.57)	1.03 (2.73)	1.36 (0.91)	66.08 (13.64)	2.41 (3.95)	1.58 (1.98)
Overweight	66.82 (14.59)	1.21 (2.66)	1.50 (1.16)	68.79 (13.04)	2.92 (4.88)	1.81 (2.60)
Obese	67.23 (14.33)	1.09 (2.69)	1.44 (1.19)	67.60 (13.99)	2.89 (3.83)	1.47 (1.12)
**Physical activity**						
Low	63.28 (15.27)	1.08 (2.72)	1.54 (1.22)	64.22 (14.40)	3.06 (5.10)	1.60 (1.73)
Moderate	64.43 (15.77)	0.87 (2.46)	1.42 (0.99)	64.67 (14.50)	2.52 (4.24)	1.66 (2.26)
High	65.29 (14.27)	1.44 (3.03)	1.39 (1.09)	66.84 (13.57)	2.62 (4.09)	1.54 (1.58)
**Tobacco use**						
Never	65.52 (15.08)	0.97 (2.49)	1.41 (0.94)	66.43 (13.99)	2.62 (4.42)	1.72 (2.28)
Current	62.88 (15.19)	1.30 (2.99)	1.40 (1.16)	64.83 (14.19)	2.55 (3.90)	1.44 (1.42)
Past	62.14 (16.35)	1.73 (3.88)	1.64 (1.42)	61.54 (14.27)	2.73 (4.12)	1.49 (1.26)
**Alcohol consumption**						
Lifetime abstainer	64.81 (15.13)	1.07 (2.64)	1.40 (1.02)	65.61 (14.12)	2.59 (4.28)	1.61 (2.05)
Non-heavy drinker	62.70 (16.41)	1.45 (3.63)	1.36 (0.93)	65.66 (14.36)	2.73 (3.48)	1.65 (1.38)
Infrequent heavy drinker	63.77 (13.87)	0.80 (1.31)	1.00 (0.00)	66.62 (20.51)	2.36 (4.56)	1.33 (0.58)
Frequent heavy drinker	63.73 (15.24)	0.87 (2.90)	2.69 (2.06)	66.02 (10.98)	2.79 (4.83)	1.27 (0.65)

* Higher WHOQoL score indicates better quality of life (on a scale of 0 to 100); SD: Standard Deviation

Table S3 WHOQoL-8 score by domains and number of disease count

A. Wave 2

Domain	0 NCD	1 NCD	2 NCDs	3+ NCDs	Total	*p*-value
Overall Quality of Life	62.72 (21.30)	60.31 (20.53)	58.27 (20.79)	56.41 (21.28)	61.07 (21.11)	< 0.001
Overall Health	71.19 (19.93)	64.25 (21.81)	59.90 (23.61)	56.16 (22.86)	66.81 (21.75)	< 0.001
Energy Levels	61.31 (25.23)	55.57 (24.62)	51.83 (24.35)	50.20 (23.44)	57.76 (25.16)	< 0.001
Ability to Perform Daily Activities	70.18 (20.41)	65.89 (20.87)	63.25 (22.89)	59.05 (23.73)	67.35 (21.37)	< 0.001
Self-esteem	72.95 (19.45)	68.99 (20.45)	65.04 (22.19)	62.36 (22.94)	70.13 (20.63)	< 0.001
Personal Relationships	73.56 (19.66)	70.70 (19.85)	69.10 (21.67)	67.86 (21.27)	71.81 (20.18)	< 0.001
Money	49.36 (26.94)	48.86 (25.52)	48.26 (25.79)	50.65 (24.93)	49.17 (26.27)	0.271
Living Conditions	73.64 (20.36)	72.52 (20.91)	72.04 (21.73)	70.72 (22.12)	72.93 (20.83)	0.001

B. Wave 3

Domain	0 NCD	1 NCD	2 NCDs	3+ NCDs	Total	*p*-value
Overall Quality of Life	66.01 (19.34)	62.34 (19.07)	61.57 (19.72)	59.38 (20.19)	63.62 (19.53)	< 0.001
Overall Health	69.11 (18.36)	62.86 (19.88)	59.40 (20.93)	55.28 (23.24)	64.49 (20.25)	< 0.001
Energy Levels	68.55 (21.38)	61.05 (22.19)	58.17 (22.62)	53.14 (22.62)	63.32 (22.55)	< 0.001
Ability to Perform Daily Activities	69.70 (17.34)	64.70 (19.76)	61.97 (20.64)	58.45 (22.89)	66.00 (19.52)	< 0.001
Self-esteem	71.74 (16.78)	67.93 (18.18)	65.52 (19.10)	63.77 (20.90)	68.92 (18.19)	< 0.001
Personal Relationships	73.17 (15.77)	69.86 (16.95)	68.28 (17.95)	66.80 (19.28)	70.85 (16.96)	< 0.001
Money	54.35 (25.21)	53.72 (24.31)	53.29 (25.07)	52.39 (24.87)	53.82 (24.89)	0.207
Living Conditions	74.94 (16.40)	73.25 (18.06)	72.37 (18.50)	72.65 (18.70)	73.83 (17.48)	< 0.001

Notes:
Values are mean (standard deviation)
p-values based on one-way ANOVA
Equal variances assessed using Bartlett's test
Domains based on WHOQoL-8
Higher WHOQoL scores indicate better quality of life (on a scale of 0 to 100)
