# Supplementary material for: Multiple long-term conditions and their association with quality of life and healthcare utilisation among adults in India: a cross-sectional analysis of WHO SAGE Waves 2 and 3
Source: BMJ Open. 2026 Aug 11;16(8):e116399. doi: 10.1136/bmjopen-2026-116399 (PMC13475192; doi:10.1136/bmjopen-2026-116399)
Supplement: Supplementary file 2 [file bmjopen-2026-116399.reviewer_comments.pdf]

PEER REVIEW HISTORY

BMJ Open publishes all reviews undertaken for accepted manuscripts. Reviewers are asked to complete a checklist review form and are provided with free text boxes to elaborate on their assessment. These free text comments are reproduced below.

ARTICLE DETAILS

Title (Provisional)

Multiple long-term conditions and their association with quality of life and healthcare utilisation among adults in India: a cross-sectional analysis of WHO SAGE Waves 2 and 3

Authors

Banstola, Amrit; Ahmed, Sayem; Shenoy, M Swathi; Gupta, Priti; Kondal, Dimple; Bhagat, Radhika; Goenka, Shifalika; Khunti, Kamlesh; Prabhakaran, Dorairaj; Mohan, Sailesh; Pokhrel, Subhash

---

VERSION 1 - REVIEW

**Reviewer**	**1**
**Name**	**Prazeres, Filipe**
**Affiliation**	**Universidade da Beira Interior Faculdade de Ciencias da Saude**
**Date**	**27-Feb-2026**
**COI**	**None**

Thank you for the invitation to review this manuscript. I appreciate the opportunity to evaluate this interesting and important contribution for multimorbidity field.

The study offers a rigorous and well-structured analysis of how specific multimorbidity patterns influence quality of life and healthcare utilization among adults aged ≥18 years in India. The results clearly demonstrate that depression coexisting with COPD or stroke is associated with the most marked reductions in overall well-being. In terms of healthcare use, the COPD–stroke combination generates the highest outpatient service demand, while depression with angina corresponds to the greatest hospitalization rates. The progressive decline in physical and functional domains of quality of life with increasing numbers of long-term conditions is convincingly presented. These findings are well supported by the analyses and are consistently articulated throughout the manuscript.

Overall, the study is methodologically sound, clinically meaningful, and relevant for health system planning and physicians' work. I recommend acceptance.

**Reviewer**	**2**
**Name**	**Polidori, M Cristina**
**Affiliation**	**Universität zu Köln**
**Date**	**19-May-2026**
**COI**	**None**

The manuscript addresses an important and highly relevant public health topic: the increasing burden of multiple long-term conditions (multimorbidity) in India and its association with quality of life and healthcare utilisation. The study is well designed, the analyses are appropriate, and the results are clearly presented. Overall, the work is of high importance and makes a valuable contribution to the literature. There are, however, issues that should be addressed.

> The interpretation that multimorbidity per se is directly associated with reduced quality of life should be refined. Evidence from the broader literature suggests that the relationship between multimorbidity and both survival and quality of life is not straightforward and is often mediated by functional status, symptom burden, psychological factors, and social determinants. The discussion would benefit from a more cautious framing of causal interpretations.

> The dataset provides an opportunity for further analyses exploring potential mediating mechanisms. In particular, selected domains from the WHO Quality of Life instrument (e.g., self-esteem, daily functioning, and physical functioning) could be examined as potential mediators in the association between multimorbidity and overall quality of life. This would substantially strengthen the conceptual contribution of the work.

> The operational definition of multimorbidity as "more than two chronic conditions" should be critically discussed. While commonly used, this threshold may be increasingly limited in its conceptual validity, particularly in younger populations and in evolving epidemiological contexts. A reflection on alternative definitions or sensitivity analyses using different cut-offs would be valuable.

> The discussion would benefit from stronger integration into the existing international literature on multimorbidity. Extensive methodological and conceptual work on multimorbidity has been carried out in the past decades including the seminal studies on multimorbidity patterns and clustering approaches are notably absent and should be incorporated to better situate the findings.

> The manuscript would benefit from more age-attuned language and a stronger emphasis on functional outcomes rather than disease counts alone. This would align the discussion more closely with contemporary frameworks such as functional ability and intrinsic capacity, which are increasingly central in ageing and chronic disease research.

VERSION 1 - AUTHOR RESPONSE

Response to reviewers

We thank both reviewers for their comments that have strengthened this manuscript. We have responded to each of the comments below.

REVIEWER 1

Prof. Filipe Prazeres, Universidade da Beira Interior Faculdade de Ciencias da Saude, CINTESIS

The study offers a rigorous and well-structured analysis of how specific multimorbidity patterns influence quality of life and healthcare utilization among adults aged ≥18 years in India. The results clearly demonstrate that depression coexisting with COPD or stroke is associated with the most marked reductions in overall well-being. In terms of healthcare use, the COPD-stroke combination generates the highest outpatient service demand, while depression with angina corresponds to the greatest hospitalization rates. The progressive decline in physical and functional domains of quality of life with increasing numbers of long-term conditions is convincingly presented. These findings are well supported by the analyses and are consistently articulated throughout the manuscript. Overall, the study is methodologically sound, clinically meaningful, and relevant for health system planning and physicians' work. I recommend acceptance.

Response:
We thank Reviewer 1 for this positive assessment and for recommending acceptance.

REVIEWER 2

Dr. M Cristina Polidori, Universität zu Köln

The manuscript addresses an important and highly relevant public health topic: the increasing burden of multiple long-term conditions (multimorbidity) in India and its association with quality of life and healthcare utilisation. The study is well designed, the analyses are appropriate, and the results are clearly presented. Overall, the work is of high importance and makes a valuable contribution to the literature. There are, however, issues that should be addressed.

Response:
We thank Reviewer 2 for the constructive and substantive comments. We have addressed each comment in turn below.

Comment 1

The interpretation that multimorbidity per se is directly associated with reduced quality of life should be refined. Evidence from the broader literature suggests that the relationship between multimorbidity and both survival and quality of life is not straightforward and is often mediated

by functional status, symptom burden, psychological factors, and social determinants. The discussion would benefit from a more cautious framing of causal interpretations.

Response:

We agree. The original text overstated the directness of the multimorbidity-quality of life relationship. We have revised the relevant passage in the Discussion to acknowledge that observed associations likely reflect mediated rather than direct effects, and to explicitly note the constraint imposed by the cross-sectional design. The revised text cites mediation analyses from the existing literature, including a multi-country SAGE analysis and longitudinal work from India, that document functional limitation, pain/discomfort, and sleep/energy as partial mediators of this relationship.

Manuscript change:

The following passage was revised in the Discussion (dose-response relationship and mediating pathways):

> A clear dose-response relationship was evident between disease count and WHOQoL-8 scores, with individuals reporting three or more conditions experiencing the largest adjusted reduction in quality of life. This pattern aligns with prior evidence reporting cumulative decrements in quality of life with each additional disease [27,59,60]. The relationship is not, however, a direct one. A mediation analysis using WHO SAGE data from six LMICs found that mobility limitations explained 47.9% of the association between physical multimorbidity and quality of life, with pain/discomfort (43.5%) and sleep/energy (35.0%) also mediating this relationship [61]. Longitudinal and mediation studies indicate that the association between MLTC (or higher disease counts) and poorer quality of life is partly explained by intermediate pathways, particularly functional limitations and health-system or socioeconomic factors [24,62,63]. The disproportionate declines observed in the physical, energy, and self-esteem domains of WHOQoL-8, set against the relative stability of the financial satisfaction domain, support this interpretation: people may experience reduced quality of life because chronic conditions constrain what they can do and how they perceive themselves, rather than as a direct consequence of disease count alone. Causal claims warrant caution, given the cross-sectional design of each wave and the likelihood that observed associations reflect mediated rather than direct effects.

Comment 2

The dataset provides an opportunity for further analyses exploring potential mediating mechanisms. In particular, selected domains from the WHO Quality of Life instrument (e.g., self-esteem, daily functioning, and physical functioning) could be examined as potential mediators in the association between multimorbidity and overall quality of life. This would substantially strengthen the conceptual contribution of the work.

Response:

We have considered this carefully. Formal mediation analysis using WHOQoL-8 domain scores as candidate mediators was not feasible in this study as it was outside the scope of this study. We have instead expanded the Discussion to explain this constraint explicitly, to draw on the domain-level results to indicate which functional pathways are most implicated, and to identify formal mediation analysis as a priority for future research.

Manuscript change:

The following passages were added to the Limitations section and to the implications for future research paragraph:

Revised text (Limitations):

> Formal mediation analysis was not undertaken, as several WHOQoL-8 items that could serve as candidate mediators are themselves components of the composite outcome.

Revised text (Implications on policy, practice, and research):

> Examining mediating mechanisms will require longitudinal data with functional status, symptom burden, and psychological wellbeing measured independently of the composite quality-of-life outcome. Cross-sectional mediation analyses using WHO SAGE data [61] and structural equation modelling with Indian survey data [24] have begun to examine how MLTC, functional limitations and socioeconomic disadvantages related to quality of life in older adults. Future surveys should incorporate independently measured functional and clinical assessments alongside WHOQoL-8 to make such analyses possible. Future surveys should also expand the range of conditions captured to enable more precise estimates of MLTC prevalence and burden.

Comment 3

The operational definition of multimorbidity as 'more than two chronic conditions' should be critically discussed. While commonly used, this threshold may be increasingly limited in its conceptual validity, particularly in younger populations and in evolving epidemiological contexts. A reflection on alternative definitions or sensitivity analyses using different cut-offs would be valuable.

Response:
We have added a substantive paragraph to the Discussion critically examining the ≥2 threshold. We acknowledge that it treats clinically heterogeneous combinations as analytically equivalent, that its meaning varies across the adult age range, and that sensitivity analyses using weighted indices were not possible given the absence of severity data in SAGE. We reference the Delphi consensus study by Ho and colleagues, which affirmed the ≥2 threshold as standard but called for greater condition standardisation. We also note that the nine-condition SAGE instrument likely understates true burden. No sensitivity analysis using alternative count-based thresholds was conducted, as the limited condition set would make such analyses analytically unhelpful.

Manuscript change:
The following paragraph was added to the Discussion (after the sociodemographic findings paragraph):

> The operational definition of MLTCs used here, defined as two or more chronic conditions, follows widespread convention but requires critical reflection. The ≥2 threshold treats hypertension co-occurring with cataracts as analytically equivalent to depression co-occurring with stroke, despite the very different consequences for daily life evident in our domain-specific results, where depression-COPD and depression-stroke dyads were associated with the lowest WHOQoL-8 scores (45.3 and 47.5 respectively). Ho and colleagues' Delphi consensus confirmed the ≥2 threshold but emphasised the need for greater standardisation [72]. The study reached agreement on 24 conditions to always include in multimorbidity measures and a further 35 to usually include. Simple counts

were preferred for prevalence and clustering, weighted measures for risk adjustment and outcome prediction. The ≥2 threshold may also carry different meanings across the age range, with two co-occurring conditions in a person aged 30 years representing a clinically distinct phenomenon from the same count in a person aged 70 years. Our use of nine conditions available from the SAGE dataset excludes several common burdens such as cardiovascular disease beyond angina, cancer, dementia, and chronic kidney disease, so estimates here probably understate true MLTC prevalence. Sensitivity analyses using weighted indices were not possible because the dataset does not capture severity, and the limited condition set restricts the analytical value of alternative count-based thresholds. Future work using linked electronic health record data or expanded survey instruments would allow a more detailed examination of how threshold and condition choice shape estimates of burden.

Comment 4

The discussion would benefit from stronger integration into the existing international literature on multimorbidity. Extensive methodological and conceptual work on multimorbidity has been carried out in the past decades including the seminal studies on multimorbidity patterns and clustering approaches are notably absent and should be incorporated to better situate the findings.

Response:

We have integrated the international multimorbidity clustering literature more substantively. The revised Discussion now references Barnett and colleagues' landmark Scottish study (1.75 million patients), the factor-analytic work by Prados-Torres and colleagues in Spanish primary care, the Ng et al. systematic review of clustering methods, and cross-national analyses including the Asian systematic review by Rajoo et al. and the harmonised ATHLOS cohort analysis by Bayes-Marin et al. The revised paragraph explicitly connects the hypertension-dominant dyads observed in our data to the cardiometabolic cluster, and the depression-COPD and depression-stroke combinations to the mental-physical cluster, as identified consistently across this international literature. We also added a further paragraph explaining why depression-inclusive combinations produce the lowest WHOQoL-8 scores, drawing on the domain structure of the instrument and supporting evidence from WHO SAGE Wave 1 data.

Manuscript change:

The following paragraph was added to the Discussion (after the sociodemographic and socioeconomic patterns paragraph):

> The dyad patterns identified in this analysis are consistent with a substantial international literature on MLTC clustering. Barnett and colleagues' landmark Scottish study of 1.75 million patients showed that physical-mental comorbidity becomes more common as both condition count and socioeconomic deprivation increase [51]. Using exploratory factor analysis in 275,682 adult patients from Spanish primary care, Prados Torres and colleagues identified five recurring multimorbidity patterns including cardiometabolic and depressive clusters [53]. Cross-national analyses, including a systematic review of eight Asian studies and harmonised cohort data spanning LMICs and high-income countries, have consistently identified cardiovascular–metabolic and mental health as the dominant clusters, alongside musculoskeletal and respiratory groupings [54–56]. Despite heterogeneity in analytical methods factor analysis, hierarchical clustering, and latent class analysis, three broad groupings have emerged consistently across settings: cardiovascular-metabolic, mental health, and musculoskeletal or respiratory conditions

[54]. A European study found three broadly consistent patterns in both Spain and the Netherlands and showed that the cardiometabolic pattern increased in complexity with age [57]. The present findings fit this picture closely. The hypertension-dominant dyads reflect the cardiometabolic cluster; the depression-COPD and depression-stroke combinations represent the mental–physical cluster in an Indian epidemiological context.

The particularly low WHOQoL-8 scores for depression-inclusive dyads (45.3 for depression–COPD; 47.5 for depression–stroke) reflect the distinct functional burden these combinations impose. Unlike largely asymptomatic conditions such as hypertension or early-stage diabetes, depression, COPD, and stroke each directly impair the domains the WHOQoL-8 measures: depression through reduced energy, self-esteem, and perceived functioning; COPD through breathlessness and activity limitation; stroke through acute multi-domain disability. Several WHOQoL-8 domains, including energy, self-esteem, and ability to perform daily activities, closely resemble core depressive symptom domains; prior work using a related instrument has noted that this content overlap may amplify depression–QoL associations [42]. Schrock and colleagues, using WHO SAGE Wave 1 data from adults in six middle-income countries, found that greater cumulative chronic morbidity was associated with higher subjective fatigue, with a clear dose-response pattern [58]. This is consistent with the energy and functional domain declines observed here.

Comment 5

The manuscript would benefit from more age-attuned language and a stronger emphasis on functional outcomes rather than disease counts alone. This would align the discussion more closely with contemporary frameworks such as functional ability and intrinsic capacity, which are increasingly central in ageing and chronic disease research.

Response:

Two key clarifications informed our response. First, this study covers adults aged ≥18 years, not exclusively older adults. Framing the manuscript primarily around ageing frameworks would misrepresent its scope. Second, ageing and intrinsic capacity frameworks remain relevant given the concentration of multimorbidity burden in later life, where prevalence in Wave 3 reached 32.3% among adults aged 60 years and older. The domain-specific WHOQoL-8 findings, particularly the marked declines in energy levels, physical functioning, and ability to perform daily activities, directly reflect the functional domains emphasised by these frameworks. We have revised the Discussion to acknowledge the age structure of the burden, to note where functional pathway findings align with the WHO intrinsic capacity framework, and to call for future age-stratified analyses. The paper has not been restructured around ageing as a primary lens, which would be inconsistent with its stated scope.

Manuscript change:

The following sentences were added to the relevant paragraph in the Discussion:

Although the analytical sample spans adults aged 18 years and above, the multimorbidity burden was heavily concentrated in later life: prevalence in Wave 3 reached 32.3% among adults aged 60 years and older, compared with 3.2% among those aged 18 to 44 years. The financial domain of WHOQoL-8 remained stable across disease counts in both waves, while the physical and functional domains overall health, energy levels, and ability to perform daily activities fell most sharply. This pattern suggests that the lived

experience of multimorbidity is shaped primarily through functional and physical pathways rather than through perceived financial strain, at least within the age range and socioeconomic context of this sample. Qualitative studies have highlighted how multimorbidity generates a multifaceted physical and functional workload, resulting in substantial burdens and disruption to everyday life [64]. Future age-stratified analyses using longitudinal Indian data would help clarify how these functional impacts vary across the adult life course. Observed declines in physical function and energy levels, alongside stable financial satisfaction, align with the WHO's intrinsic capacity framework, which frames healthy ageing in terms of functional ability rather than the mere absence of disease [65].

Note: The 11 new references added are highlighted in the reference section of the revised manuscript.

VERSION 2 - REVIEW

**Reviewer**	**2**
**Name**	**Polidori, M Cristina**
**Affiliation**	**Universität zu Köln**
**Date**	**22-Jul-2026**
**COI**	

My points have been adequately considered and integrated.
